# Intragenic Locus in Human *PIWIL2* Gene Shares Promoter and Enhancer Functions

**DOI:** 10.1371/journal.pone.0156454

**Published:** 2016-06-01

**Authors:** Yulia V. Skvortsova, Sofia A. Kondratieva, Marina V. Zinovyeva, Lev G. Nikolaev, Tatyana L. Azhikina, Ildar V. Gainetdinov

**Affiliations:** Department of Genomics and Postgenomic Technologies, Shemyakin-Ovchinnikov Institute of Bioorganic Chemistry, Russian Academy of Sciences, Moscow, Russia; University of Naples Federico II, ITALY

## Abstract

Recently, more evidence supporting common nature of promoters and enhancers has been accumulated. In this work, we present data on chromatin modifications and non-polyadenylated transcription characteristic for enhancers as well as results of *in vitro* luciferase reporter assays suggesting that *PIWIL2* alternative promoter in exon 7 also functions as an enhancer for gene *PHYHIP* located 60Kb upstream. This finding of an intragenic enhancer serving as a promoter for a shorter protein isoform implies broader impact on understanding enhancer-promoter networks in regulation of gene expression.

## Introduction

Regulation of gene expression is a multilayer process, which involves such aspects as promoters, enhancers, transcription factors, chromatin modifications, and spatial organization of the nucleus. All these constituents interact through crosstalk and their complex interplay results in different expression patterns across a range of tissues and cell types [1–3]. Nevertheless, recent advances in studies of transcription regulation revealed common features specific to sites of active gene expression: colocalization of transcribed genes in transcription factories [4, 5], interaction of several enhancers with several promoters inside topologically associating domains (TADs) [6], similar chromatin modifications within TADs [7–10], transcription initiated at enhancers (enhancer RNA, eRNA) [11, 12]. Another emerging insight is the common architecture of promoters and enhancers as platforms for initiation of transcription leading to relatively stable protein-coding mRNA and transient eRNA, respectively [13–15]. These findings point at the fact that enhancers could potentially act as promoters and vice versa, given the right cellular environment. Among such previously reported instances are enhancers of murine alpha-globin locus located in introns of gene *Nprl3* which function as alternative promoters for this gene giving rise to new mRNA products [16]. However, no alternative protein products were detected in this case. Further genome-wide transcriptome analysis revealed that up to 50% of intragenic enhancers initiate transcription of eRNA and almost 10% produce alternative variants of mRNA for the corresponding genes in erythroid cells in mice [16]. Additionally, between 1% and 7.5% of active transcription start sites (TSS) have apparent enhancer chromatin modifications in human cell lines [16].

In this work, we report a set of both indirect and direct evidence to demonstrate a similar setting for the human testis-specific gene *PIWIL2*, which is a central player in piRNA/PIWI pathway responsible for epigenetic silencing of retrotransposons [17, 18]. Specifically, the previously described alternative promoter of *PIWIL2* in exon 7 appears to be able to act as an enhancer for the gene *PHYHIP* located 60 Kb upstream. Although *PHYHIP* is highly expressed in brain tissues [19] and was suggested to play a role in the development of neurological abnormalities observed in Down syndrome patients [20], it is also co-expressed with *PIWIL2* in testis tissues [21, 22]. Taken together, this observation implies that a switch between enhancer and promoter functions could impact both mRNA and protein expression of some genes.

## Methods and Materials

### Ethics statement

Seven pairs of testicular germ cell tumor samples and corresponding adjacent normal testicular parenchyma were obtained from orchiectomy specimens. The samples were immediately frozen in liquid nitrogen. All patients provided written informed consent according to the federal law, and the study was approved by the ethical committees of the Shemyakin-Ovchinnikov Institute of Bioorganic Chemistry of the Russian Academy of Sciences and Blokhin Russian Cancer Research Center after reviewing patients’ consent and information forms.

### Cell lines

Cell lines used in experiments included TERA1 (ATCC HTB-105, testicular embryonal carcinoma [23]), NT2/D1 (ATCC CRL-1973, pluripotent testicular embryonal carcinoma [24]) and A549 (ATCC CCL-185, lung carcinoma [25]). Cells cultures were purchased from ATCC (USA) and grown in DMEM/F12 (1:1) (Invitrogen, USA) supplemented with 10% FCS (Invitrogen, USA). TCam-2 cell line [26] was kindly provided by Prof. Dr. Huebert Schorle (Department of Developmental Pathology, Institute of Pathology, Bonn Medical School, Germany). TCam-2 cells were cultured in RPMI 1640 (Invitrogen, USA) supplemented with 10% FCS (Invitrogen, USA).

### Chromatin immunoprecipitation

ChIP was performed as described earlier [27, 28] using antibodies to human histone modifications listed in S1 Table. DNA was purified using QIAquick PCR Purification Kit (Qiagen, USA). qPCR was performed using qPCRmix-HS SYBR system (Evrogen, Russia) on Lightcycler 480 (Roche, USA) in accordance with the manufacturers’ instructions. DNA fragments were amplified for 40 cycles of 95°C for 20 s, 60°C for 20 s, 72°C for 20 s. Relative level of chromatin modification was quantified with “input control” serving as the reference. Biological and technical duplicates were used to ensure reproducibility. Primer pairs used in amplification are listed in S2 Table.

### qRT-PCR

Total RNA extraction and purification from cell lines, TGCTs and normal testis samples was performed with Trizol (Thermo Scientific, USA) according to manufacturer’s instructions. First strand cDNA synthesis was carried out with either random hexanucleotide (Promega, USA) or oligo-dT primer (5’-dT_20_V-3’) and MintReverse Transcriptase (Evrogen, Russia) according to the manufacturers’ protocols. qRT-PCR reactions were performed using qPCRmix-HS SYBR system (Evrogen, Russia) on Lightcycler 480 (Roche, USA) in accordance with the manufacturers’ instructions. DNA fragments were amplified for 40 cycles of 95°C for 20 s, 60°C for 20 s, 72°C for 20 s. Relative level of mRNA was quantified with beta-actin (gene *ACTB*) mRNA serving as the reference. Technical triplicates were used to ensure reproducibility. Primer pairs used in amplification are listed in S3 Table.

### Luciferase reporter vectors, transfection and reporter gene assay

Genomic DNA was extracted from one of the normal testis sample with Wizard Genomic DNA Purification Kit (Promega, USA). Genomic regions were amplified using primers listed in S4 Table. PCR products were ligated into pGL4.10[luc2], pGL4.13[luc2/SV40] or pGL4.10mP2 (S1 File) with T4 DNA Ligase (Thermo Scientific, USA): as promoters–between NheI and BglII sites, as enhancers–in either orientation in NotI site. Prior to transfection, the constructs were linearized with PvuI or SalI restriction enzymes for forward and reverse orientation, respectively. “No enhancer” controls were also linearized with the same restriction enzymes. Transfections were performed using Lipofectamine 2000 (Invitrogen, USA) as recommended by the manufacturer. Cells were lysed 24 hours after the transfection and the activity of both firefly and *Renilla reniformis* luciferases was assessed using DualLuciferase Reporter Assay System (Promega, USA) and Tecan GENios Pro Luminometer (MTX Lab Systems, USA) according to the manufacturers’ protocols. Biological and technical duplicates were used to ensure reproducibility.

## Results and Discussion

### *In silico* evidence for the regions around *PIWIL2* exons 5 and 7 acting as enhancers

PIWIL2 is a testis-specific protein involved in piRNA pathway, which regulates expression of retrotransposons in spermatogenesis [17]. Ye *et al* have previously reported alternative promoters of *PIWIL2* gene, which they identified computationally [29]. Our group has also mapped alternative transcription start sites in *PIWIL2* exons 5 and 7 in testicular cancer cell lines TERA1 and NT2D1 using conventional molecular biology approaches [30].

Importantly, alternative promoters in exons 5 and 7 were also identified in non-testicular tissues by FANTOM5 consortium in their CAGE experiments (S1 Fig) [31]. Furthermore, additional evidence was found while analyzing chromatin state tracks based on 127 samples in the Roadmap Epigenomics Project [32]: region around *PIWIL2* exon 7 harbors epigenetic marks of a promoter in 10 primary and 4 imputed datasets (S2 and S3 Figs, respectively). Overall, these findings provide additional support for our discoveries of *PIWIL2* alternative promoters in testicular cancer cell lines.

However, closer examination of the Roadmap Epigenomics project data revealed that between 6 and 28 *primary* datasets (S2 Fig) and between 44 and 55 *imputed* datasets (S3 Fig) displayed combinations of epigenetic marks around *PIWIL2* exons 1, 5 and 7 that are characteristic for enhancers. Moreover, ENCODE data also suggests that *PIWIL2* alternative promoters both in exons 5 and 7 could function as enhancers [33]. Specifically, peaks of H3K4me1 were found in ENCODE data sets for both Tier 1 and Tier 2 cell lines (S4 and S5 Figs). Additionally, binding sites for proteins typically associated with enhancers, such as CTCF [34, 35] and P300 [36], are also located adjacent to the alternative promoters in exons 5 and 7 (S4 Fig) [37–39]. Furthermore, there is a range of putative transcription factor binding motifs around these promoters (S5 Fig) and a significant number of both tissue-specific and ubiquitously expressed transcription factors bound in various cell lines according to ENCODE ChIP-seq experiments (S5 Fig).

We additionally studied ENCODE genome segmentation data tracks which were generated for 6 cell lines using different techniques (S4 Fig, [40–43]): at least one technique assigned the region around promoter in exon 7 as an enhancer for all 6 cell lines and for 2 cell lines in case of promoters in exons 1 and 5, respectively (S4 Fig).

Altogether, there is significant amount of evidence to suggest that *PIWIL2* promoters in exons 5 and 7 could also function as enhancers. In order to examine whether this is the case in testicular tissues and cell lines, we looked at three properties of enhancers: eRNA production (associated with some enhancers), specific chromatin modifications and ability to increase promoter activity irrespective of orientation [43–47].

### Chromatin modifications around *PIWIL2* alternative promoter in exon 7 are characteristic for active enhancers

Whole-genome studies have attributed certain chromatin modifications to enhancer elements: H3K4me1/H3K27ac for active enhancers and H3K4me1/H3K27me3 for poised/inactive enhancers, unlike H3K4me3 for promoters [48]. To see whether these features are present at *PIWIL2* canonical promoter in exon 1 and alternative promoters in exons 5 and 7, we performed ChIP experiments with antibodies to these modifications on three testicular cancer related cell lines (TCam-2 –seminoma derived, TERA1 and NT2D1 –embryonal carcinoma derived) and one lung carcinoma cell line (A549). The promoter of *GAPDH* (housekeeping gene) was used as a positive control and *PIWIL2* intron 8 as well as an intergenic locus on chromosome 1 as negative controls.

**Fig 1:**
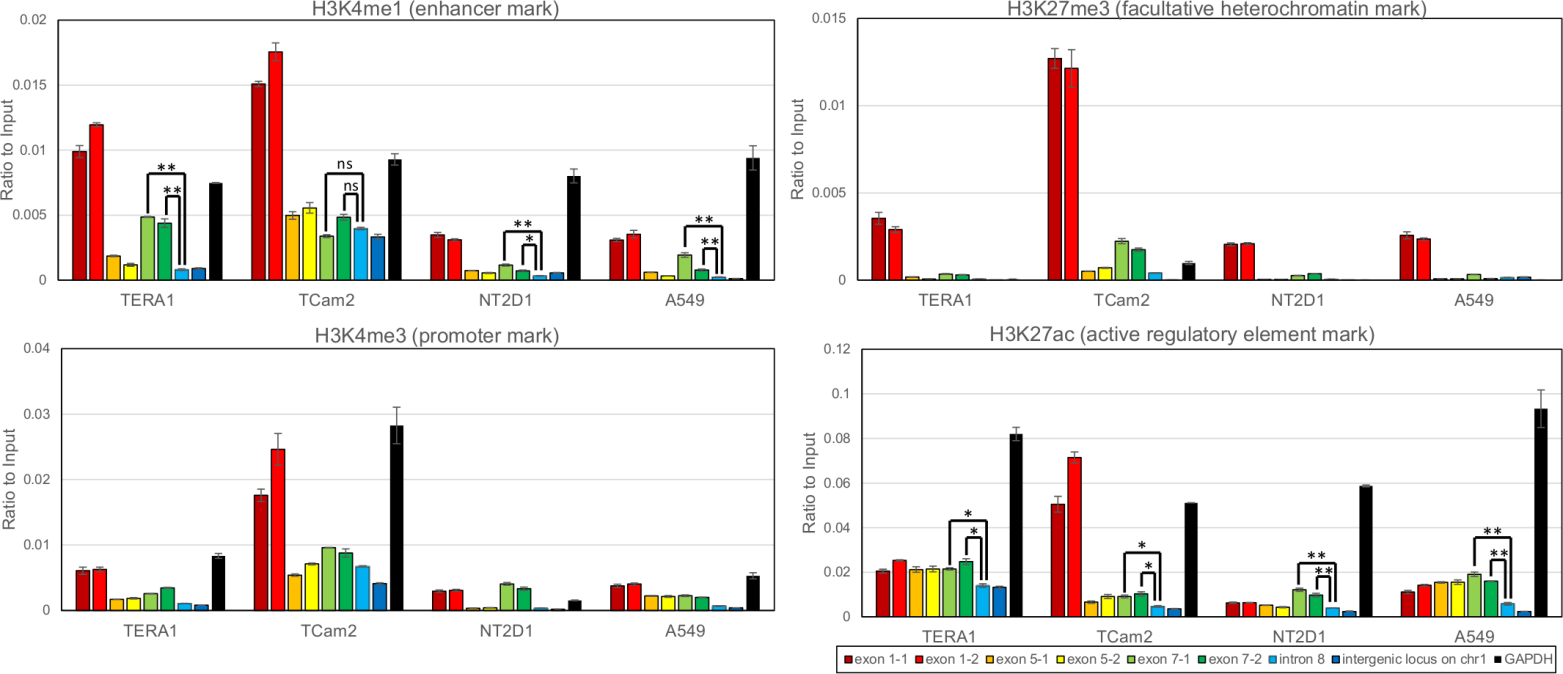
Chromatin modifications around *PIWIL2* canonical and alternative promoters. Relative level of H3K4me1 (Histone 3 lysine 4 monomethylated, enhancer mark), H3K4me3 (Histone 3 lysine 4 trimethylated, active promoter mark), H3K27ac (Histone 3 lysine 27 acetylated, active regulatory element mark) and H3K27me3 (Histone 3 lysine 27 trimethylated, facultative heterochromatin mark) histone modifications assessed by ChIP-PCR with two sets of primer pairs around *PIWIL2* canonical promoter in exon 1 and alternative promoters in exons 5 and 7. Results are are shown for four cell lines: TERA1 and NT2D1 –embryonal carcinoma, TCam2 –seminoma, and A549 –lung carcinoma. The negative controls are *PIWIL2* intron 8 and an intergenic locus on chromosome 1, the positive control is *GAPDH* promoter. P-value summary of Mann-Whitney non-paired U test is presented for some peaks (ns–non-significant, *—p-value<0.05, **—p-value<0.01).

Interestingly, though *PIWIL2* canonical promoter in exon 1 harbors histone modifications characteristic both for promoters (H3K4me3) and for enhancers (H3K4me1) in all cell lines tested, it is likely to be inactive due to the presence of the silencing H3K27me3 mark (Fig 1). Lack of promoter activity at exon 1 is also supported by previous findings showing that *PIWIL2* is expressed as its N-truncated protein isoforms in the majority of cell lines and cancer tissues [29, 30].

Further, unlike the genomic region around exon 5, the alternative promoter in exon 7 demonstrates statistically significant enrichment of H3K4me1 mark in comparison with the negative controls in all cell lines except TCam-2 (Fig 1). Moreover, the level of H3K27ac marks around exon 7 is also higher than in the negative control (Fig 1), which suggests that it could be an actively functioning enhancer, at least, in TERA1, NT2D1 and A549 cell lines.

### *PIWIL2* alternative promoters in exons 5 and 7 produce abundant non-polyadenylated transcripts

Recently, some enhancers were found to be transcribed by Pol II and produce eRNA (enhancer RNA), which are either polyadenylated or non-polyadenylated short transcripts (both spliced and non-spliced) arising around enhancers uni- or bidirectionally [49]. In one of the first studies reporting non-polyadenylated eRNA production, these enhancer associated transcripts were identified by analyzing total RNA [50]. Therefore, we attempted to use a similar approach and assessed the presence of non-polyadenylated transcription around *PIWIL2* alternative promoters using qRT-PCR on total RNA and compared it with results on its polyA+ fraction. We designed 15 primer pairs evenly covering the whole length of the *PIWIL2* gene and targeting specifically exon-exon junctions in order to minimize the contribution of possible residual genomic DNA in samples. The results were normalized to the efficiency of each primer pair and a ratio of total RNA to polyA+ fraction was calculated. This way we established the transcriptional profile along the gene and the contribution of non-polyadenylated transcripts at each point along *PIWIL2* gene.

**Fig 2:**
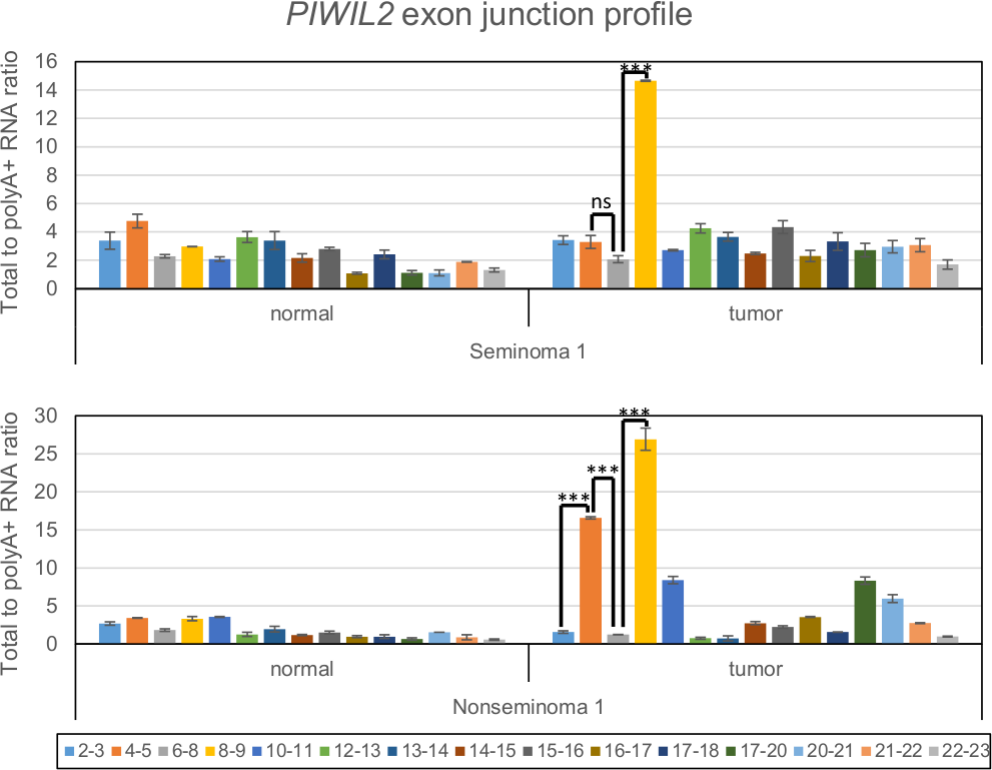
Non-polyadenylated transcription across exon-exon junctions of *PIWIL2* gene. qRT-PCR was used to assess the level of total RNA and its polyA+ fraction and the ratio of total RNA to polyA+ fraction was calculated. Seminoma and nonseminoma testicular cancer samples as well as adjacent normal testis tissues were assayed. P-value summary of Mann-Whitney non-paired U test is presented for 4–5 and 8–9 exon-exon junction peaks (ns–non-significant, ***—p-value<0.001).

Results for 7 normal/tumor pairs of testicular cancer samples (both seminomatous and nonseminomatous) are quite heterogeneous (Fig 2 and S6 Fig), which is consistent with previously published data indicating that both normal testis tissues and testicular cancers feature significant variability among individuals [51]. However, the data clearly demonstrate that all tumor samples and some adjacent normal testis tissues feature prominent and statistically significant peaks for some exon junctions, particularly 4–5 and 8–9 (Fig 2 and S6 Fig). A less pronounced but similar picture was observed for TERA1, NT2D1 and A549 cell lines (S7 Fig). This finding apparently points at the presence of a significant fraction of non-polyadenylated transcripts around exons 5 and 7, which could be the eRNA transcripts.

### Luciferase reporter assays demonstrate that *PIWIL2* alternative promoter in exon 7 can function as an enhancer for *PHYHIP* gene promoter

**Fig 3:**
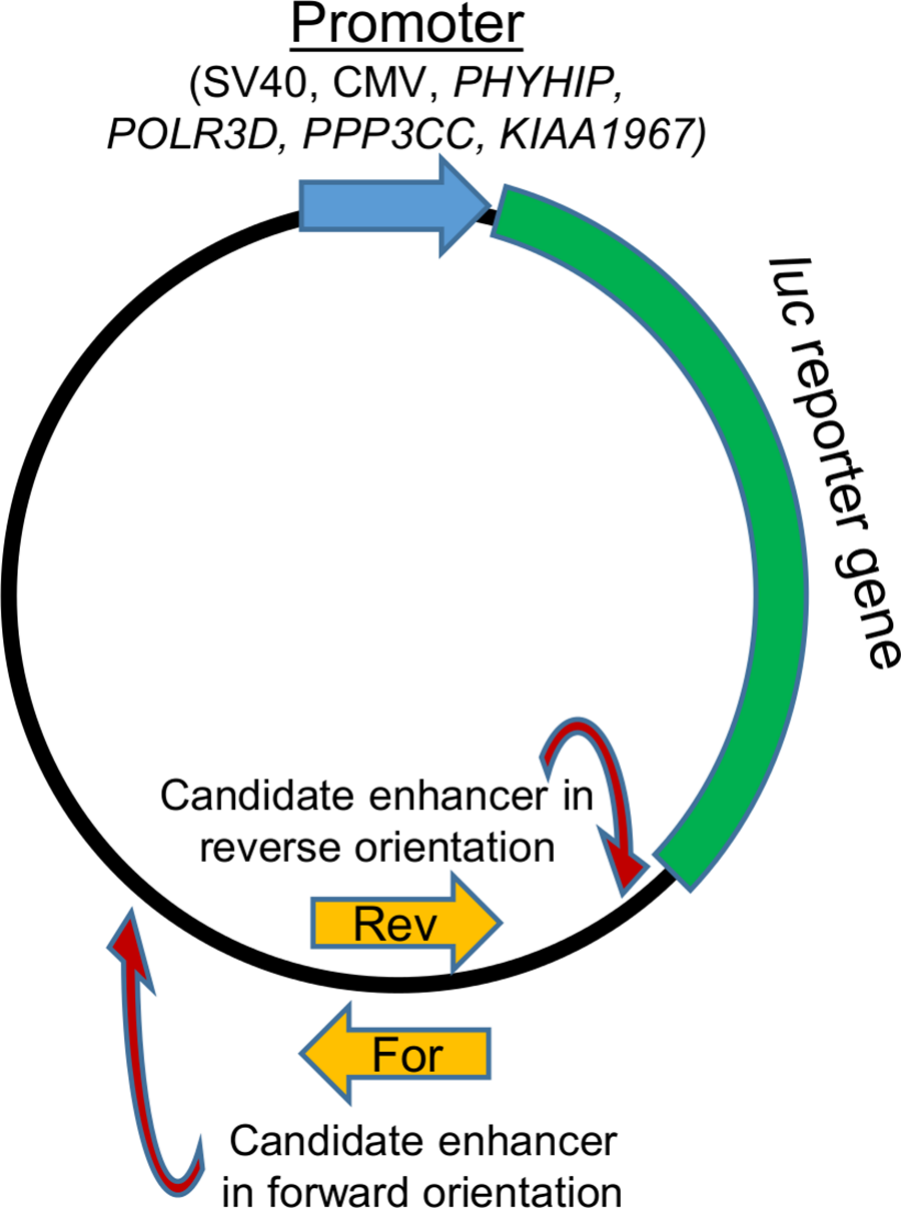
Structure of luciferase reporter vectors used in the assays. pGL4.10 plasmid was used to construct vectors with *luc* gene expression driven by various promoters (upper part in brackets) and a candidate enhancer in either orientation (lower part). Because the candidate enhancers also possess promoter activity, to discern between those, the vectors were linearized by cutting at the sites shown with red arrows. Note that after such linearization the candidate enhancer will be located either upstream (reverse orientation) or downstream (forward orientation) of the *luc* gene and its promoter.

Although chromatin modifications and eRNA production are regarded as indirect evidence, the essential property of an enhancer is to increase activity of a promoter in cell type-specific manner [47]. To test whether *PIWIL2* canonical and alternative promoters are able to act as enhancers, we cloned them in both orientations into luciferase reporter vectors downstream of the *luc* gene driven by either SV40 or CMV promoter (Fig 3, S5 Fig and S4 Table) and equimolar quantities of the constructs were probed in two cell lines (TERA1 and NT2D1). Importantly, the genomic regions, which we tested for enhancer properties, were previously shown to possess promoter activity as well. Therefore, in order to distinguish between promoter and enhancer properties, we linearized the luciferase reporter vectors by cutting them downstream (forward orientation) or upstream (reverse orientation) of the site where we placed the candidate enhancers under study (Fig 3). Importantly, after linearization the candidate enhancer will be located either downstream (forward orientation) or upstream (reverse orientation) of the luciferase reporter gene with the promoter (Fig 3). It allows us to test another aspect of enhancer function: ability to increase promoter’s activity regardless of its position (upstream or downstream).

**Fig 4:**
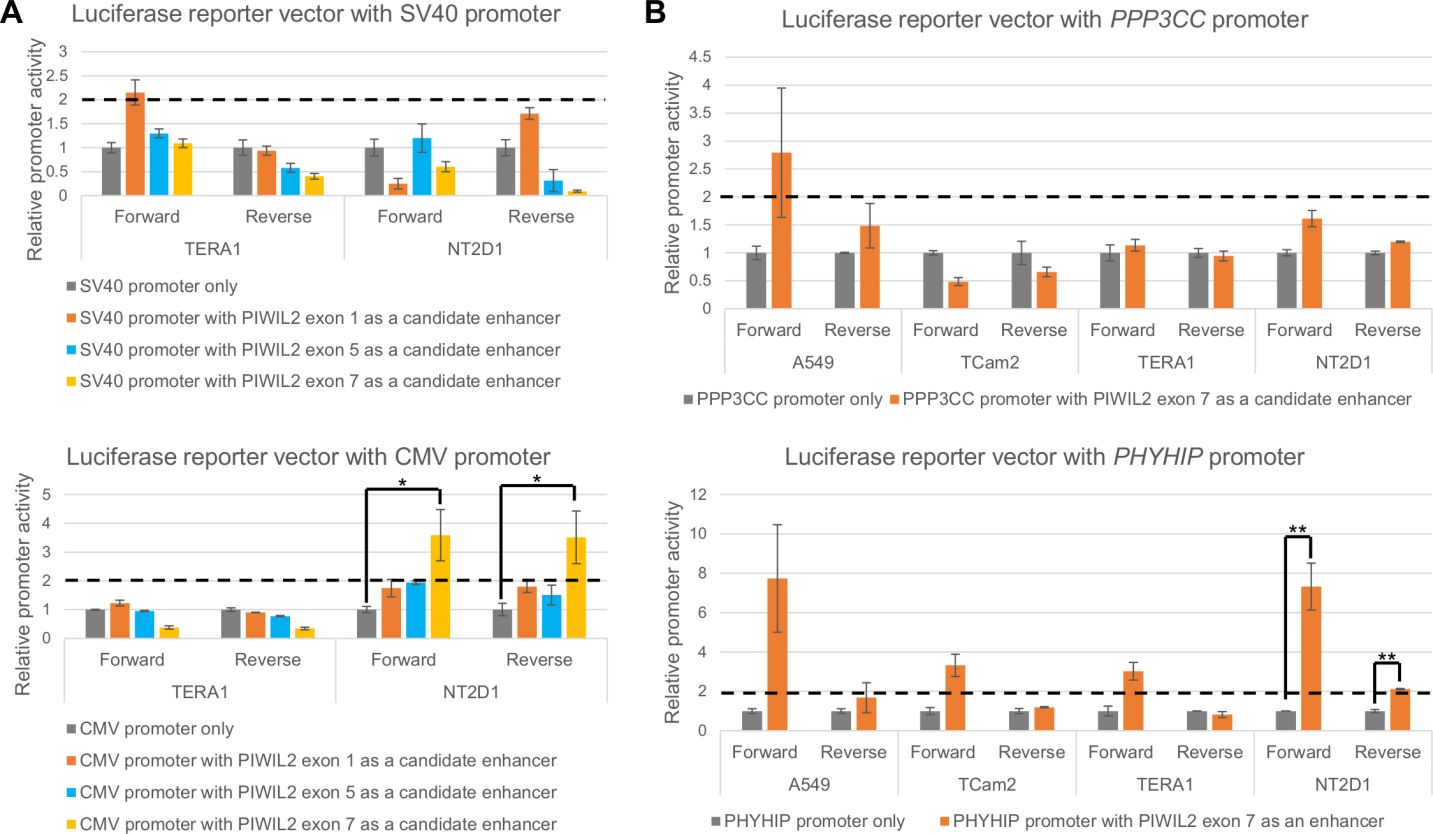
*PIWIL2* alternative promoter in exon 7 acts as an enhancer in luciferase reporter constructs. Relative promoter activity of SV40 and CMV promoters was assessed in luciferase reporter vectors from Fig 3 in two cell lines: TERA1 and NT2D1 (embryonal carcinoma, panel A). *PPP3CC* and *PHYHIP* (panel B) promoters were assessed in luciferase reporter vectors from Fig 3 in four cell lines: TERA1 and NT2D1 –embryonal carcinoma, Tcam2 –seminoma, and A549 –lung carcinoma. P-value summary of Mann-Whitney non-paired U test is presented for peaks showing more than two-fold increase (above horizontal dashed lines) for both forward and reverse orientation of the candidate enhancer compared to “no enhancer” controls (*—p-value<0.05, **—p-value<0.01).

Interestingly, more than two-fold increase of the activity of only CMV promoter and exclusively in NT2D1 cell line was detected for *PIWIL2* genomic region around exon 7 (Fig 4A), which confirms its ability to act as an enhancer.

We further wanted to find an *in vivo* target of this enhancer/alternative promoter in *PIWIL2* exon 7. As most enhancers exert their activity within about 100Kb and typically inside a topologically associating domain (TAD) [52], we looked at a recently generated Kilobase-resolution Hi-C map of genomic interactions (S8 Fig, [53]). *PIWIL2* is located within the boundaries of a contact domain also containing genes *POLR3D* (RNA polymerase III subunit [54]) and *PHYHIP* (Phytanoyl-CoA 2-Hydroxylase Interacting Protein [19]) (S8 Fig). Luciferase reporter vectors with *luc* gene expression driven by these promoters (S9 Fig) and the genomic region around *PIWIL2* exon 7 as a candidate enhancer were designed. Two more control constructs with the candidate enhancer and *luc* expression driven by promoters of two genes situated outside *PIWIL2* containing contact domain were also used (S8 and S9 Figs): *KIAA1967* (Cell Cycle And Apoptosis Regulator 2 [55]) and *PPP3CC* (testis-specific serine/threonine-protein phosphatase [56]).

Surprisingly, *PIWIL2* alternative promoter in exon 7 increased activity by more than two-fold of only *PHYHIP* promoter (Fig 4B), but not *POLR3D* and the two controls, which exemplifies promoter-specific action of enhancers–one of their intrinsic features. Notably, we could also observe that this enhancer activity was stronger in case of forward orientation. In fact, such instances have been reported earlier and were attributed to the possibility that one or more discrete *cis*-regulatory elements within the enhancer could confer such orientation dependence [57, 58]. Nevertheless, relying on the most conservative definition of an enhancer, which is increasing promoter activity regardless of its orientation, we could claim that *PIWIL2* alternative promoter in exon 7 is able to function as an enhancer only for *PHYHIP* promoter.

We also examined Polymerase II ChIA-PET data (Chromatin Interaction Analysis by Paired-End Tag Sequencing [59]) from ENCODE [60, 61] and found evidence that *PIWIL2* alternative promoter in exon 7 and *PHYHIP* promoter interact in human K562 cell line (K562 Pol2 ChIA-PET Interactions Rep 1 from ENCODE/GIS-Ruan, chr8:22086341..22087865-chr8:22143618..22145421,2). Although TAD structure is relatively stable across cell types, development stages and even organisms [62–64], the specific interaction between *PHYHIP* promoter and *PIWIL2* exon 7 and, in particular, its dynamics should be further explored in follow-up experiments.

## Conclusion

Altogether, the data presented here provide both indirect (chromatin modifications and eRNA) and direct (luciferase reporter assays) evidence suggesting that genomic area around exon 7 of *PIWIL2* gene acts as an enhancer for *PHYHIP* promoter. As a byproduct of eRNA transcription, this intragenic enhancer also produces an alternative *PIWIL2* mRNA isoform, which, in turn, is translated into a new PIWIL2 protein isoform [30]. Importantly, this short PIWIL2 isoform devoid of N-terminal domain exerts different properties and could promote tumorigenesis unlike its full-length counterpart ([29] and our unpublished data). Although further experiments are necessary to dissect the details and dynamics of PIWIL2 canonical and alternative promoters function, to our knowledge, this is the first report of a regulatory element being transcribed and, concomitantly, giving rise to an mRNA template for an alternative protein isoform.

From a broader perspective, this finding unveils a new form of interplay between intragenic enhancers and gene expression. Possible shift in genomic enhancer landscape in disease could contribute to deregulation of cellular processes through generating novel protein isoforms [65, 66]. Whole-genome and proteome studies are required to provide answers to these questions.

## Supporting Information

S1 Fig FANTOM5/ENCODE CAGE data around PIWIL2 gene. PIWIL2 gene transcript variants are shown as green horizontal bars in the upper panel (equal to Gencode annotations: ENST00000356766.6, ENST00000521356.1, ENST00000454009.2, ENST00000519884.1). FANTOM5 CAGE peaks are depicted as arrows in the middle panel (green–sense strand, purple–antisense strand) and accompanied by either the number of the peak (e.g., p1@PIWIL2) or its exact genomic coordinates (e.g., p@chr8:22140624–22140625). ENCODE CAGE raw signal is shown in the lower panel in TMP (CAGE tags per million reads).
(PDF)

S2 Fig The NIH Roadmap Epigenomics Mapping Consortium Data. Chromatin state learning using ChromHMM, which is based on a multivariate Hidden Markov Model. Core 15-state model (5 marks, 127 epigenomes) based on primary data around PIWIL2 gene.
(PPTX)

S3 Fig The NIH Roadmap Epigenomics Mapping Consortium Data. Chromatin state learning using ChromHMM, which is based on a multivariate Hidden Markov Model. 25-state model (12 marks, 127 epigenomes) based on imputed data around PIWIL2 gene exons 1–14.
(PPTX)

S4 Fig Chromatin segmentation based on ENCODE datasets on histone modifications, open chromatin data and specific TF binding data. Using two different unsupervised machine learning techniques (ChromHMM and Segway), the genome was automatically segmented into disjoint segments. A consensus unified segmentation (Combined) was also generated by reconciling results from the individual segmentations. Layered track of H3K4me1 chromatin modification in ENCODE Tier 1 and Tier 2 cell lines (upper part) and transcription factor ChIP-seq (lower part) are also presented.
(PPTX)

S5 Fig Canonical and alterative promoter regions of PIWIL2 used in luciferase reporter vector assays. UCSC Genome Browser view of promoter regions (marked with red arrows) along with layered tracks of H3K4me1, H3K27ac and H3K4me3 chromatin modifications in ENCODE Tier 1 and Tier 2 cell lines (upper part), DNaseI hypersensitivity clusters, transcription factor ChIP-seq and putative transcription factor binding sites (middle part), as well as raw H3K4me1, H3K4me3, H3K27ac and H3K27me3 ChIP-seq signals for K562, HeLa-S3 and NT2D1 cell lines from ENCODE (lower part, two experiments for K562 cell line performed at different laboratories are shown).
(PPTX)

S6 Fig Non-polyadenylated transcription across exon-exon junctions of *PIWIL2* gene in cancer samples. qRT-PCR was used to assess the level of total RNA and its polyA+ fraction and the ratio of total RNA to polyA+ fraction was calculated. Seminoma and nonseminoma testicular cancer samples as well as adjacent normal testis tissues were assayed.
(PDF)

S7 Fig Non-polyadenylated transcription across exon-exon junctions of *PIWIL2* gene in cell lines. qRT-PCR was used to assess the level of total RNA and its polyA+ fraction and the ratio of total RNA to polyA+ fraction was calculated. Four cell lines were assayed: TERA1 and NT2D1 –embryonal carcinoma, TCam2 –seminoma, and A549 –lung carcinoma.
(PDF)

S8 Fig Contact domains around PIWIL2 from the 1 kb resolution Hi-C map (Rao et al, 2014). The heatmap of the genomic contacts along chr8:21,880,000–22,470,000 (hg19) is presented. The contact domains are depicted as yellow boxes and the positions of *PHYHIP*, *POLR3D*, *PIWIL2* genes belonging to the same contact domain are shown. *PPP3CC* and *KIAA1967* are also presented in the neighboring contact domain.
(PDF)

S9 Fig Promoter regions of PHYHIP, POLR3D, PPP3CC and KIAA1967 used in luciferase reporter vector assays. UCSC Genome Browser view of promoter regions (marked with red arrows) along with layered tracks of H3K4me3 chromatin modification in ENCODE Tier 1 and Tier 2 cell lines (active promoter mark, upper part of each panel) and DNaseI hypersensitivity clusters (lower part of each panel).
(PPTX)

S1 File Sequence of pGL4.10mP2 construct based on pGL4.10[luc2] with CMV promoter driving expression of *luc* gene. (GB)

S1 Table Antibodies used for ChIP-PCR. (XLSX)

S2 Table Primers used for qPCR to quantitate the level of histone marks. (XLSX)

S3 Table Primers used in qRT-qPCR. immunoprecipitation. (XLSX)

S4 Table Primers used for cloning genomic regions corresponding to *PIWIL2* canonical promoter in exon 1, *PIWIL2* alternative promoters in exons 5 and 7, and promoters of genes *PHYHIP*, *POLR3D*, *PPP3CC and KIAA1967*. (XLSX)
