# Supplementary material for: Intragenic Locus in Human PIWIL2 Gene Shares Promoter and Enhancer Functions
Source: PLoS One. 2016 Jun 1;11(6):e0156454. doi: 10.1371/journal.pone.0156454 (PMC4889060; doi:10.1371/journal.pone.0156454)

FANTOM5/ENCODE CAGE data around *PIWIL2* gene

PIWIL2 gene transcript variants are shown as green horizontal bars in the upper panel (equal to Gencode annotations: ENST00000356766.6, ENST00000521356.1, ENST00000454009.2, ENST00000519884.1). FANTOM5 CAGE peaks are depicted as arrows in the middle panel (green – sense strand, purple – antisense strand) and accompanied by either the number of the peak (e.g., p1@PIWIL2) or its exact genomic coordinates (e.g., p@chr8:22140624-22140625). ENCODE CAGE raw signal is shown in the lower panel in TMP (CAGE tags per million reads).

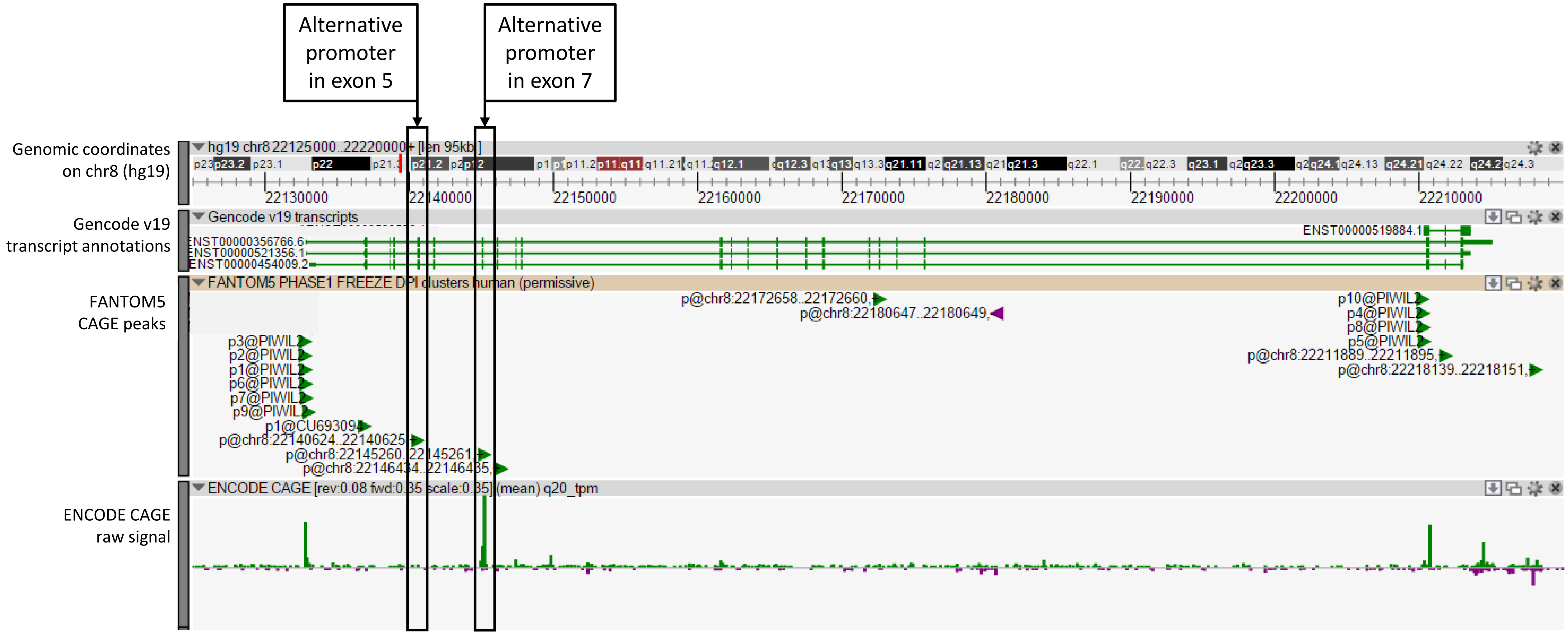

Supplement: S1 Fig — PIWIL2 gene transcript variants are shown as green horizontal bars in the upper panel (equal to Gencode annotations: ENST00000356766.6, ENST00000521356.1, ENST00000454009.2, ENST00000519884.1). FANTOM5 CAGE peaks are depicted as arrows in the middle panel (green–sense strand, purple–antisense strand) and accompanied by either the number of the peak (e.g., p1@PIWIL2) or its exact genomic coordinates (e.g., p@chr8:22140624–22140625). ENCODE CAGE raw signal is shown in the lower panel in TMP (CAGE tags per million reads). (PDF) [file pone.0156454.s001.pdf]
