# Supplementary material for: Intragenic Locus in Human PIWIL2 Gene Shares Promoter and Enhancer Functions
Source: PLoS One. 2016 Jun 1;11(6):e0156454. doi: 10.1371/journal.pone.0156454 (PMC4889060; doi:10.1371/journal.pone.0156454)
Supplement: S2 Fig — Chromatin state learning using ChromHMM, which is based on a multivariate Hidden Markov Model. Core 15-state model (5 marks, 127 epigenomes) based on primary data around PIWIL2 gene. (PPTX) [file pone.0156454.s002.pptx]

## Slide 1
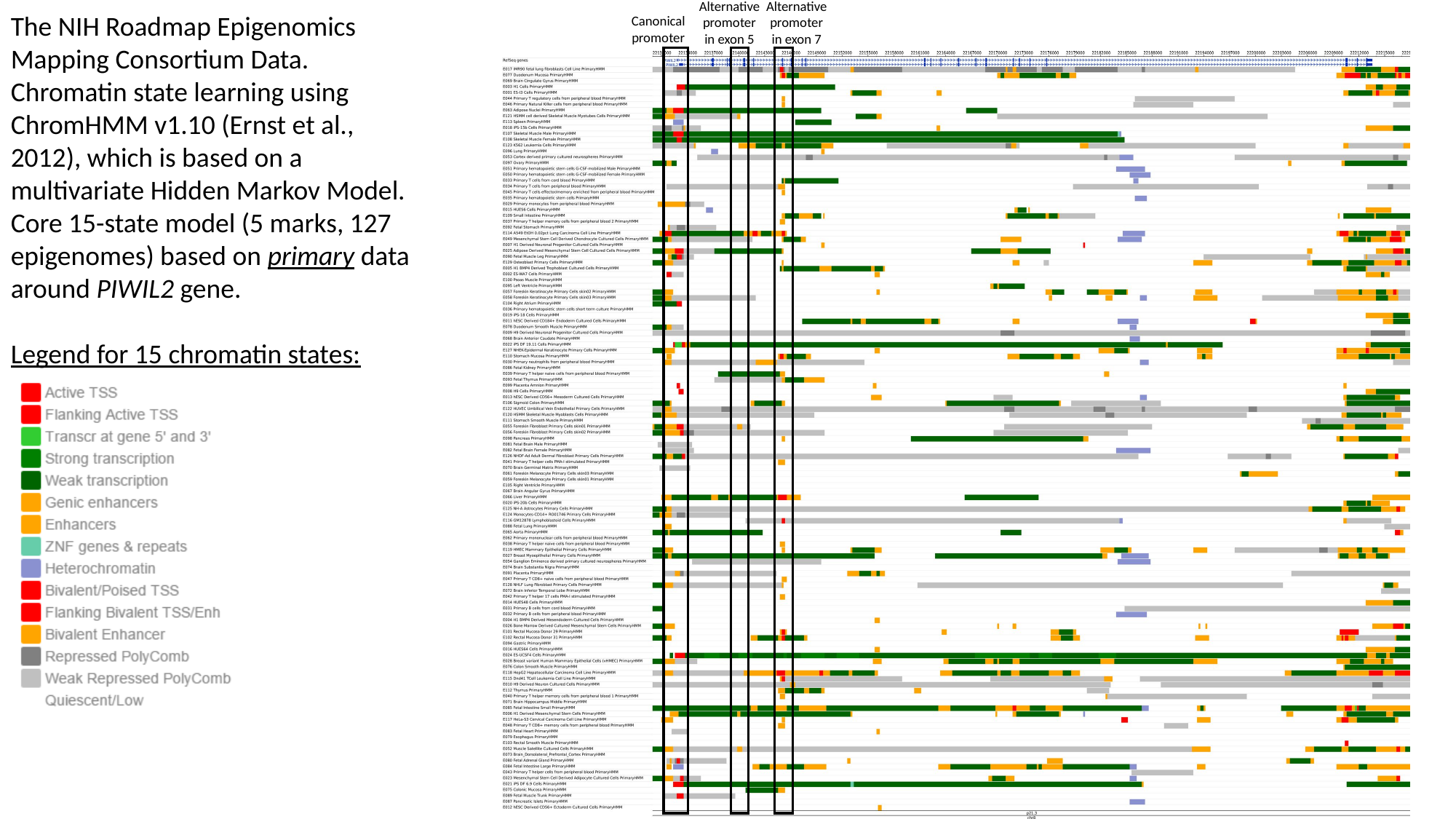

Alternative promoter in exon 5
Alternative promoter in exon 7
The NIH Roadmap Epigenomics Mapping Consortium Data. Chromatin state learning using ChromHMM v1.10 (Ernst et al., 2012), which is based on a multivariate Hidden Markov Model. Core 15-state model (5 marks, 127 epigenomes) based on primary data around PIWIL2 gene.
Legend for 15 chromatin states:
Canonical promoter
