# Supplementary material for: Intragenic Locus in Human PIWIL2 Gene Shares Promoter and Enhancer Functions
Source: PLoS One. 2016 Jun 1;11(6):e0156454. doi: 10.1371/journal.pone.0156454 (PMC4889060; doi:10.1371/journal.pone.0156454)
Supplement: S4 Fig — Using two different unsupervised machine learning techniques (ChromHMM and Segway), the genome was automatically segmented into disjoint segments. A consensus unified segmentation (Combined) was also generated by reconciling results from the individual segmentations. Layered track of H3K4me1 chromatin modification in ENCODE Tier 1 and Tier 2 cell lines (upper part) and transcription factor ChIP-seq (lower part) are also presented. (PPTX) [file pone.0156454.s004.pptx]

## Slide 1
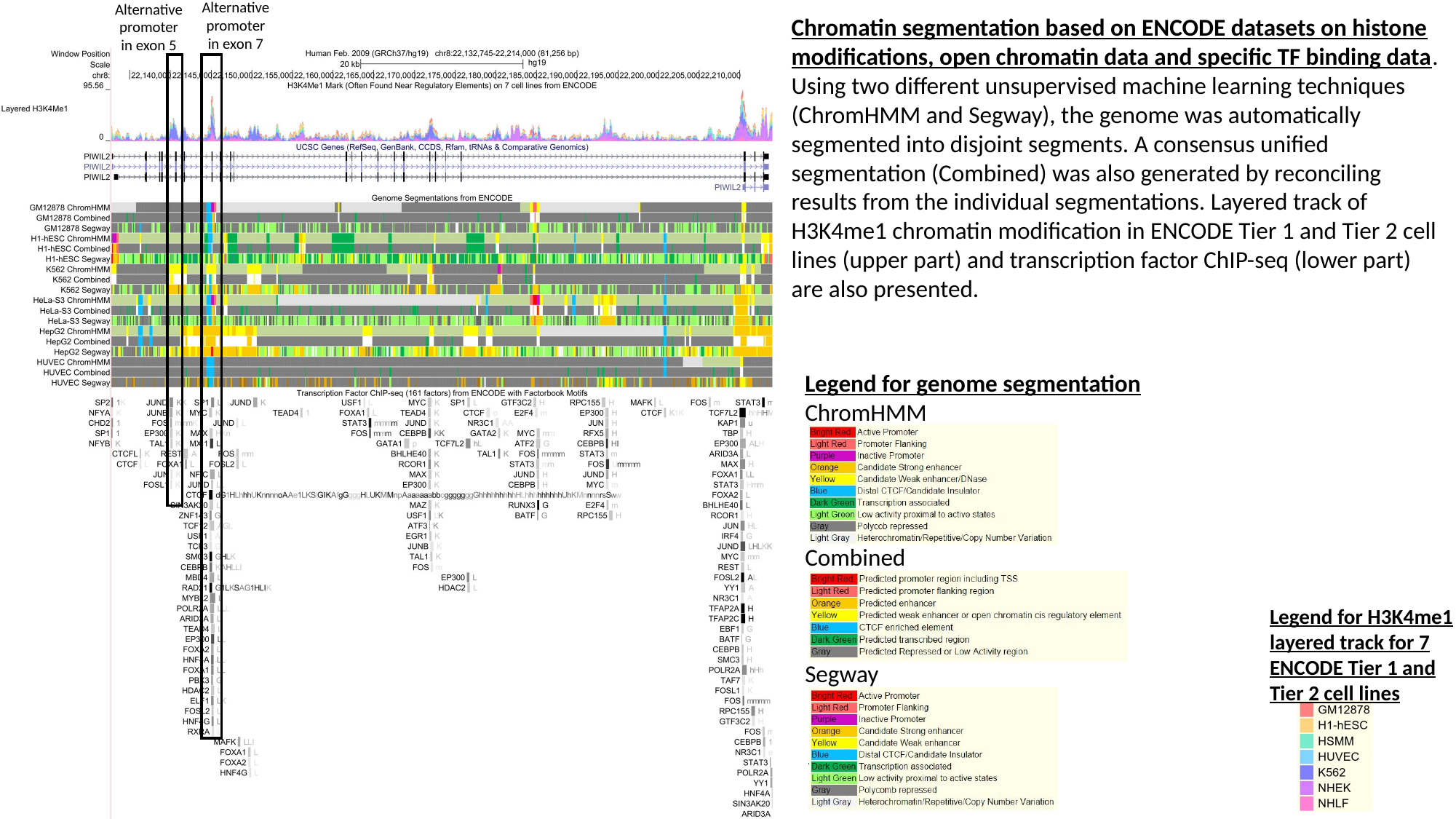

Alternative promoter in exon 7
Alternative promoter in exon 5
Chromatin segmentation based on ENCODE datasets on histone modifications, open chromatin data and specific TF binding data. Using two different unsupervised machine learning techniques (ChromHMM and Segway), the genome was automatically segmented into disjoint segments. A consensus unified segmentation (Combined) was also generated by reconciling results from the individual segmentations. Layered track of H3K4me1 chromatin modification in ENCODE Tier 1 and Tier 2 cell lines (upper part) and transcription factor ChIP-seq (lower part) are also presented.
Legend for genome segmentation
ChromHMM
Combined
Segway
Legend for H3K4me1 layered track for 7 ENCODE Tier 1 and Tier 2 cell lines
