# Supplementary material for: Intragenic Locus in Human PIWIL2 Gene Shares Promoter and Enhancer Functions
Source: PLoS One. 2016 Jun 1;11(6):e0156454. doi: 10.1371/journal.pone.0156454 (PMC4889060; doi:10.1371/journal.pone.0156454)
Supplement: S5 Fig — UCSC Genome Browser view of promoter regions (marked with red arrows) along with layered tracks of H3K4me1, H3K27ac and H3K4me3 chromatin modifications in ENCODE Tier 1 and Tier 2 cell lines (upper part), DNaseI hypersensitivity clusters, transcription factor ChIP-seq and putative transcription factor binding sites (middle part), as well as raw H3K4me1, H3K4me3, H3K27ac and H3K27me3 ChIP-seq signals for K562, HeLa-S3 and NT2D1 cell lines from ENCODE (lower part, two experiments for K562 cell line performed at different laboratories are shown). (PPTX) [file pone.0156454.s005.pptx]

## Slide 1
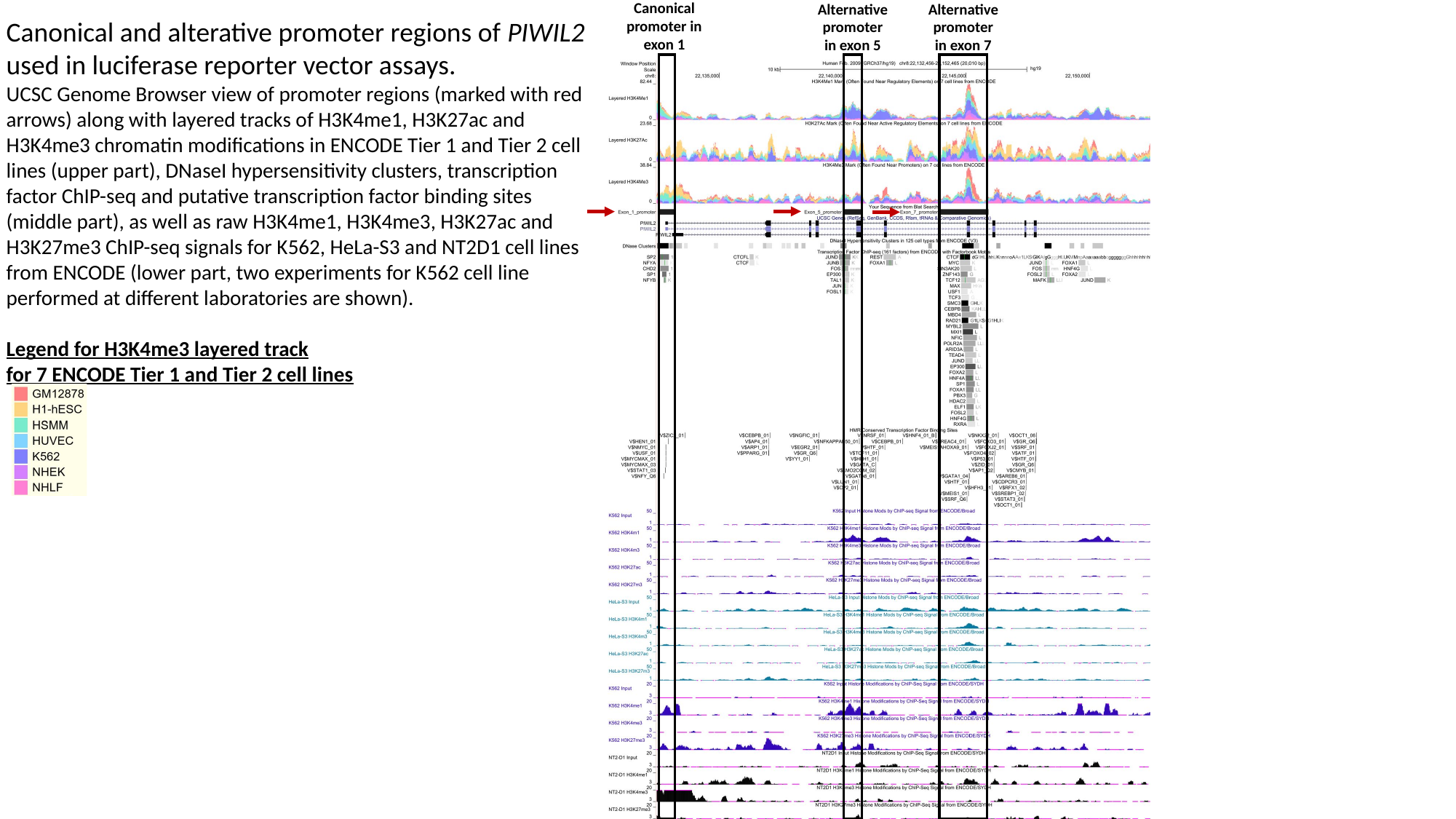

Canonical promoter in exon 1
Alternative promoter in exon 5
Alternative promoter in exon 7
Canonical and alterative promoter regions of PIWIL2 used in luciferase reporter vector assays.
UCSC Genome Browser view of promoter regions (marked with red arrows) along with layered tracks of H3K4me1, H3K27ac and H3K4me3 chromatin modifications in ENCODE Tier 1 and Tier 2 cell lines (upper part), DNaseI hypersensitivity clusters, transcription factor ChIP-seq and putative transcription factor binding sites (middle part), as well as raw H3K4me1, H3K4me3, H3K27ac and H3K27me3 ChIP-seq signals for K562, HeLa-S3 and NT2D1 cell lines from ENCODE (lower part, two experiments for K562 cell line performed at different laboratories are shown).
Legend for H3K4me3 layered track
for 7 ENCODE Tier 1 and Tier 2 cell lines
