# Supplementary figures and images for: Intragenic Locus in Human PIWIL2 Gene Shares Promoter and Enhancer Functions
Source: PLoS One. 2016 Jun 1;11(6):e0156454. doi: 10.1371/journal.pone.0156454 (PMC4889060; doi:10.1371/journal.pone.0156454)

### PIWIL2 exon junction profiles in testicular cancer samples

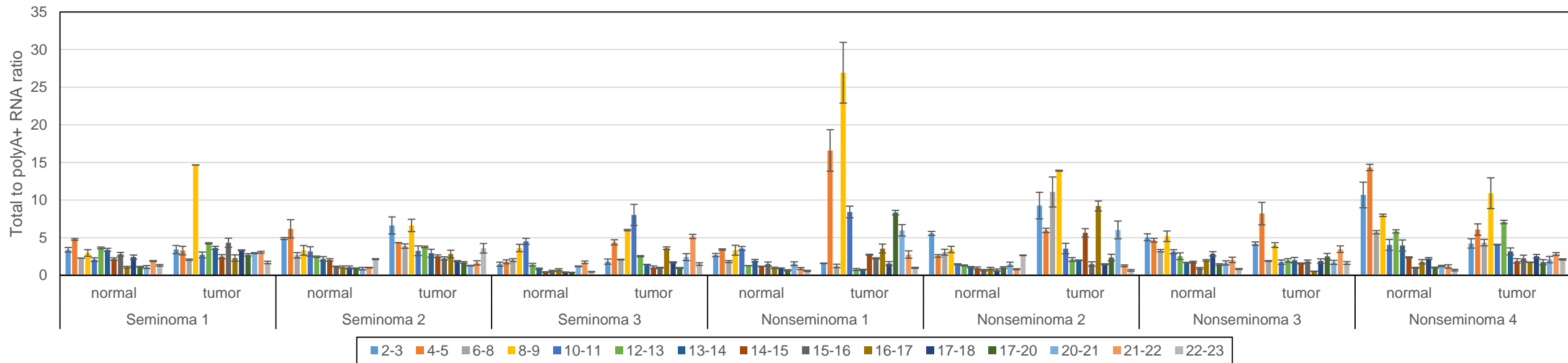

Supplement: S6 Fig — qRT-PCR was used to assess the level of total RNA and its polyA+ fraction and the ratio of total RNA to polyA+ fraction was calculated. Seminoma and nonseminoma testicular cancer samples as well as adjacent normal testis tissues were assayed. (PDF) [file pone.0156454.s006.pdf]

*PIWIL2* exon junction profiles in cell lines

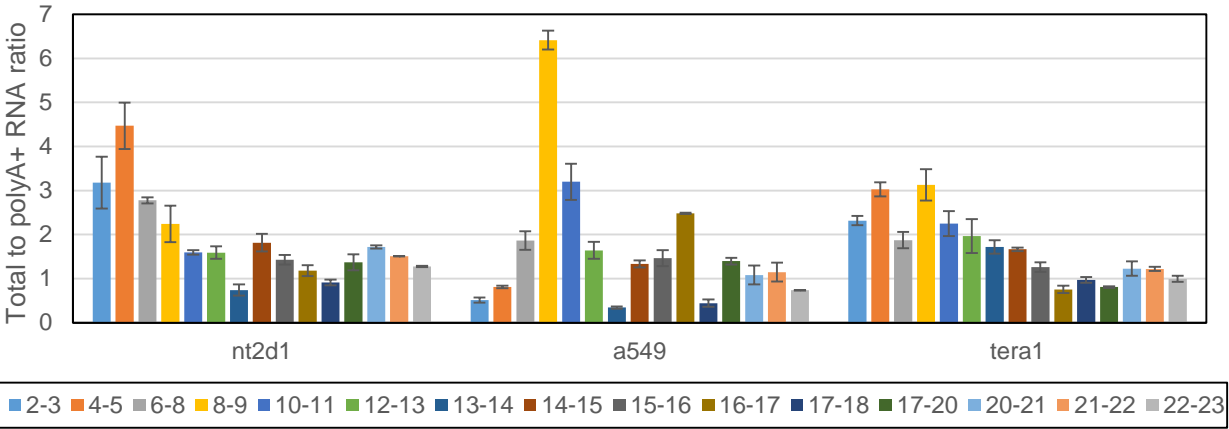

Supplement: S7 Fig — qRT-PCR was used to assess the level of total RNA and its polyA+ fraction and the ratio of total RNA to polyA+ fraction was calculated. Four cell lines were assayed: TERA1 and NT2D1 –embryonal carcinoma, TCam2 –seminoma, and A549 –lung carcinoma. (PDF) [file pone.0156454.s007.pdf]
