# Supplementary material for: Intragenic Locus in Human PIWIL2 Gene Shares Promoter and Enhancer Functions
Source: PLoS One. 2016 Jun 1;11(6):e0156454. doi: 10.1371/journal.pone.0156454 (PMC4889060; doi:10.1371/journal.pone.0156454)

Contact domains around *PIWIL2* from the 1 kb resolution Hi-C map (Rao *et al.*, Cell, 159(7):1665-1680, 2014).

The heatmap of the genomic contacts along chr8:21,880,000-22,470,000 (hg19) is presented. The contact domains are depicted as yellow boxes and the positions of *PHYHIP, POLR3D, PIWIL2* genes belonging to the same contact domain are shown. *PPP3CC* and *KIAA1967* are also presented in the neighboring contact domain.

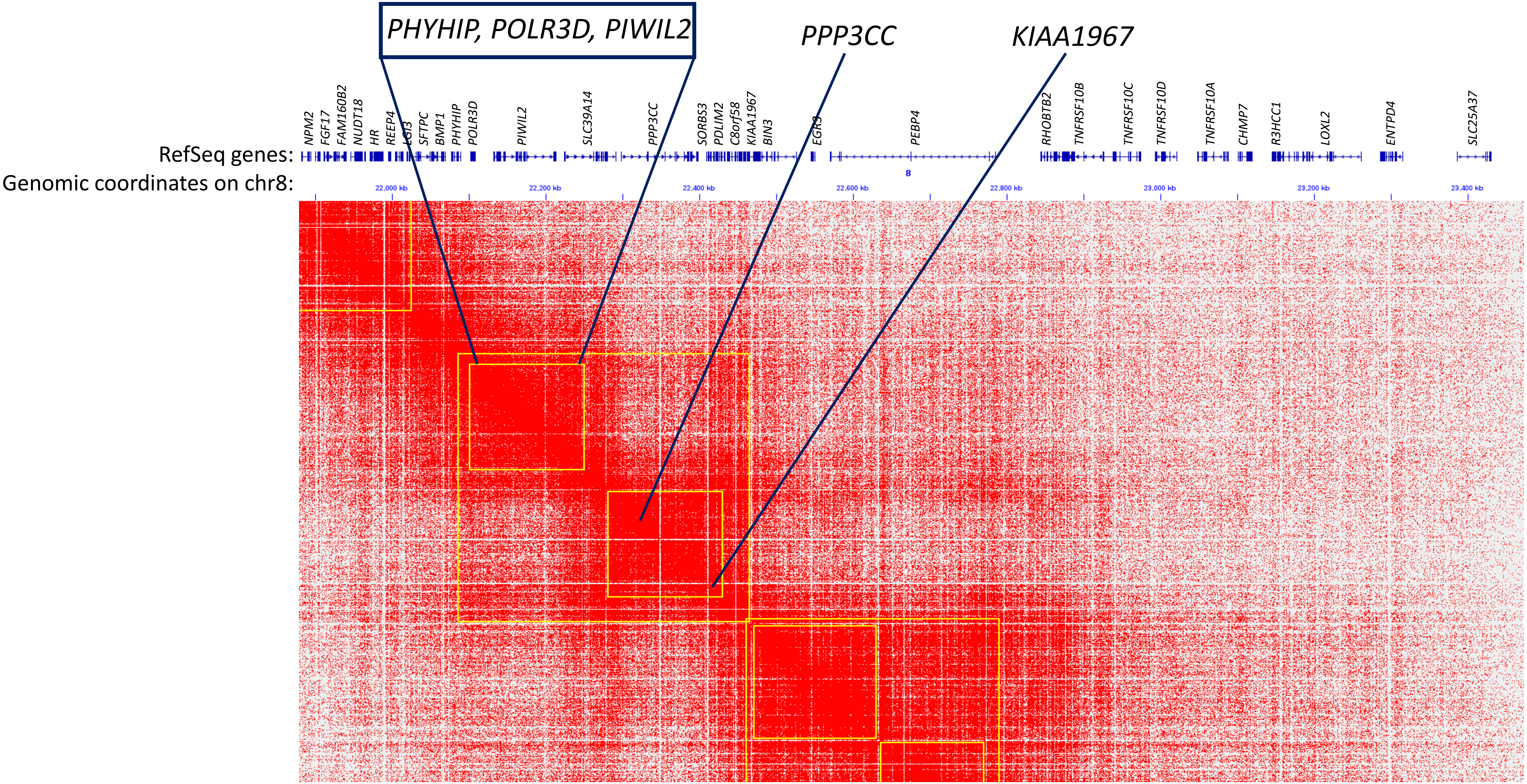

Supplement: S8 Fig — The heatmap of the genomic contacts along chr8:21,880,000–22,470,000 (hg19) is presented. The contact domains are depicted as yellow boxes and the positions of PHYHIP, POLR3D, PIWIL2 genes belonging to the same contact domain are shown. PPP3CC and KIAA1967 are also presented in the neighboring contact domain. (PDF) [file pone.0156454.s008.pdf]
