# Supplementary material for: Intragenic Locus in Human PIWIL2 Gene Shares Promoter and Enhancer Functions
Source: PLoS One. 2016 Jun 1;11(6):e0156454. doi: 10.1371/journal.pone.0156454 (PMC4889060; doi:10.1371/journal.pone.0156454)
Supplement: S9 Fig — UCSC Genome Browser view of promoter regions (marked with red arrows) along with layered tracks of H3K4me3 chromatin modification in ENCODE Tier 1 and Tier 2 cell lines (active promoter mark, upper part of each panel) and DNaseI hypersensitivity clusters (lower part of each panel). (PPTX) [file pone.0156454.s009.pptx]

## Slide 1
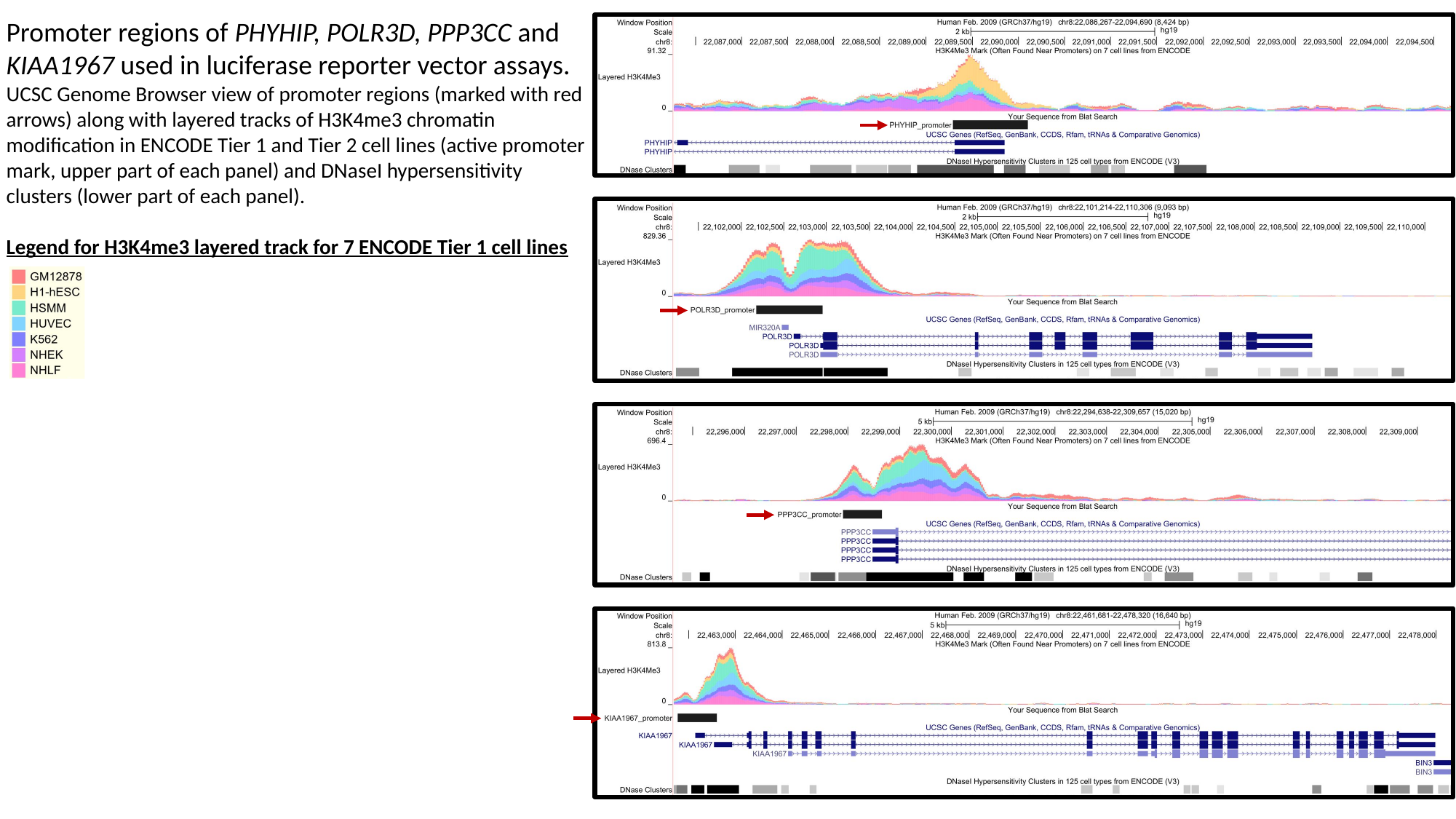

Promoter regions of PHYHIP, POLR3D, PPP3CC and KIAA1967 used in luciferase reporter vector assays.
UCSC Genome Browser view of promoter regions (marked with red arrows) along with layered tracks of H3K4me3 chromatin modification in ENCODE Tier 1 and Tier 2 cell lines (active promoter mark, upper part of each panel) and DNaseI hypersensitivity clusters (lower part of each panel).
Legend for H3K4me3 layered track for 7 ENCODE Tier 1 cell lines
